# Generalised Anxiety Disorder – A Twin Study of Genetic Architecture, Genome-Wide Association and Differential Gene Expression

**DOI:** 10.1371/journal.pone.0134865

**Published:** 2015-08-14

**Authors:** Matthew N. Davies, Serena Verdi, Andrea Burri, Maciej Trzaskowski, Minyoung Lee, John M. Hettema, Rick Jansen, Dorret I. Boomsma, Tim D. Spector

**Affiliations:** 1 Twin Research and Genetic Epidemiology, King’s College London, London, United Kingdom; 2 Translational Oncogenomics Laboratory, Centre for Evolution and Cancer, Division of Molecular Pathology, The Institute of Cancer Research, London, United Kingdom; 3 Social Genetic & Developmental Psychiatry, Institute of Psychiatry, Psychology & Neuroscience, King’s College London, London, United Kingdom; 4 Virginia Institute for Psychiatric and Behavioral Genetics, Department of Psychiatry, Virginia Commonwealth University, Richmond, VA, United States of America; 5 Department of Biological Psychology, VU University Amsterdam, Neuroscience Campus, Amsterdam, the Netherlands; 6 Department of Psychiatry, VU University Medical Center, Neuroscience Campus, Amsterdam, the Netherlands; The George Washington University, UNITED STATES

## Abstract

Generalised Anxiety Disorder (GAD) is a common anxiety-related diagnosis, affecting approximately 5% of the adult population. One characteristic of GAD is a high degree of anxiety sensitivity (AS), a personality trait which describes the fear of arousal-related sensations. Here we present a genome-wide association study of AS using a cohort of 730 MZ and DZ female twins. The GWAS showed a significant association for a variant within the RBFOX1 gene. A heritability analysis of the same cohort also confirmed a significant genetic component with h2 of 0.42. Additionally, a subset of the cohort (25 MZ twins discordant for AS) was studied for evidence of differential expression using RNA-seq data. Significant differential expression of two exons with the ITM2B gene within the discordant MZ subset was observed, a finding that was replicated in an independent cohort. While previous research has shown that anxiety has a high comorbidity with a variety of psychiatric and neurodegenerative disorders, our analysis suggests a novel etiology specific to AS.

## Introduction

Generalised Anxiety Disorder (GAD) is a chronic disorder characterised by persistent worrying, anxiety symptoms, and tension independent of recent stressful events. GAD is one of the most prevalent anxiety disorders affecting approximately 5% of the adult population and is twice as common in women as in men [1,2]. Anxiety symptoms are frequently combined with an assortment of somatic and psychological complaints, such as sleep disruption, irritability, autonomic arousal, restlessness, fatigue and difficulties in concentrating. GAD often presents with comorbid disorders such as Major Depression Disorder, Bipolar Disorder and substance abuse [3] but it is an independent disorder which is described within the DSM-5[4]. A strong aspect of GAD is a high level of anxiety sensitivity (AS), which is defined as the fear of arousal related sensations, arising from beliefs that these anxiety-related sensations have harmful consequences [5–7]. While it is unclear whether AS represents a “lower order facet of trait anxiety” or rather a distinct construct [8], current research suggests that anxiety sensitivity may usefully be conceptualised as a variable risk factor for anxiety-related problems [9]. Previous research has suggested that heightened AS may contribute to mechanisms underlying GAD, particularly the expression and processing of emotional cognitions [10,11].

A commonly used clinical metric to measure AS is the Anxiety Sensitivity Index (ASI)[12], which quantifies overall psychological and somatic symptoms related to anxiety [13–15]. The ASI is based upon a 16-item self-report questionnaire assessing a person’s beliefs about the social and somatic consequences of anxiety symptoms. The questions are designed to assess the subject’s fear of physical symptoms, fear of publicly observable anxious symptoms and fear of cognitive dyscontrol [16]. Previous studies using the ASI have repeatedly demonstrated that patients with GAD have a higher ASI score compared to healthy controls [17] and that the physical, social and cognitive facets of ASI are all indicators of clinical GAD [16,17]. The ASI has been shown to have strong internal consistency (a = 0.81–0.94), a good degree of test/retest reliability (r = 0.71–0.75), and a high degree of inter-item relatedness [13,14,18]. While MZ twins arise from the same single cell and therefore share a near identical genome, they show phenotypic discordance for many complex traits and disorders including autism (58 to 60%) [19] and schizophrenia (58%) [20]. This observation suggests that many complex traits, have a strong non-genetic component. The discordant MZ model is a powerful tool in order to identify non-genetic contributions to a phenotype including variation in gene expression not mediated by cis- or trans-eQTL effects[21]. Here we present a twin study design to identify genetic associations with AS, as well as a differential gene expression analysis of MZ twins who are discordant for AS.

## Materials/Methods

### Sample Collection

AS data was collected from participants previously recruited from the TwinsUK registry [22,23]. The 730 participants comprised 143 MZ and 222 DZ twin pairs with an age range of 38–84 years. The TwinsUK cohort has previously been shown to be comparable to population singletons in terms of disease-related and a range of lifestyle characteristics [24,25]. The participants completed a detailed health questionnaire which included the ASI questionnaire (see S1 Table). Data on potential confounding factors such as age, BMI, smoking and alcohol consumption were also collected as part of the study. Peripheral blood samples were collected, and LCLs were generated by EBV transformation of the B-lymphocyte component by the European Collection of Cell Cultures agency[26]. The St. Thomas' Research Ethics Committee (REC) approved on 20 September 2007 the protocol for the dissemination of data, including DNA, with REC reference number RE04/015. On 12 March 2008, the St Thomas' REC confirmed that this approval extended to expression data. Volunteers gave informed consent and signed an approved consent form before the biopsy procedure. Volunteers were supplied with an appropriate detailed information sheet regarding the research project.

### Heritability calculation

A heritability analysis of the cohort of 730 MZ and DZ female twins was conducted with the structural equation modeling package Mx[27]. The goodness of fit of the genetic models was evaluated by comparing them with an unconstrained saturated model, which estimates the maximum number of parameters without partitioning variance into genetic and environmental components. This gives rise to a likelihood ratio chi-square tests(-2LL; significant tests indicate significant deterioration in fit) and Akaike’s Information Criterion (AIC; a parsimony fit index, with lower values indicating the more suitable model)[28]. The best-fitting models were selected on the basis of parsimony.

### Genotyping and GWAS

Genotyping of the cohort of 730 MZ and DZ female twins was carried out with a combination of Illumina arrays (HumanHap300, HumanHap610Q, 1M-Duo, and 1.2MDuo 1M). Intensity data for each of the three arrays were pooled separately (with 1M-Duo and 1.2MDuo 1M pooled together), and genotypes were called with the Illuminus calling algorithm with the use of a threshold on a maximum posterior probability of 0.95. Samples were imputed into the 1000 Genomes Phase 1 reference panel (data freeze, 10/11/2010) [29] using IMPUTE2 [30] and filtered (MAF<0.05, IMPUTE info value < 0.8)[26].

**Fig 1:**
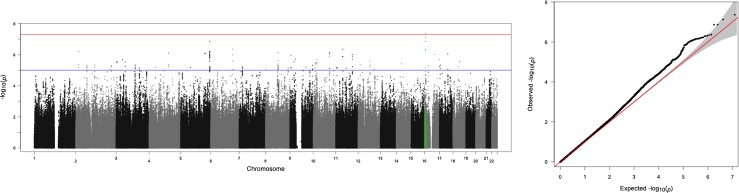
a:b: Manhattan and qq plot of ASI GWAS with RBFOX1 gene region highlighted. (a) The rs13334105 SNP (p values 4.39 x 10–8) highlighted in green is the only variant to show genomewide significant association with ASI. (b) The qq plot of ASI GWAS.

A GWAS analysis on the full cohort of twins using AS as the phenotype was implemented using the program GEMMA [31]. The analysis used age as a covariate and also incorporated the family structure of the twins (see Fig 1A and 1B, Table 1).

**Table 1:** Top associations of ASI GWAS, showing several SNPs of suggestive significance within the RBFOX1 coding region. rs13334105, the only variant of genomewide significant association is shown in bold.

Chr	Position	Gene	β-value	S.E.	p-value	rs number	Polymorphism	MAF
**16**	**6203620**	**RBFOX1**	**-8.99E+00**	**1.64E+00**	**4.39E-08**	**rs13334105**	**T/G**	**5.164**
16	6209696	RBFOX1	-8.74E+00	1.63E+00	7.57E-08	rs7186032	T/C	5.04
5	174297685	-	-3.69E+00	7.01E-01	1.36E-07	rs9313698	T/G	7.562
16	6211100	RBFOX1	-8.48E+00	1.61E+00	1.39E-07	rs73529988	T/G	5.082
6	132561425	-	-7.26E+00	1.44E+00	4.34E-07	rs9402394	A/C	9.366
11	42058503	RP11-148I19.1	-3.79E+00	7.51E-01	4.45E-07	rs10837768	G/A	11.252
16	6175152	RBFOX1	-8.79E+00	1.75E+00	4.87E-07	rs77368405	A/G	8.877
16	6179301	RBFOX1	-8.77E+00	1.75E+00	5.11E-07	rs60610784	G/A	8.892
16	6178043	RBFOX1	-8.76E+00	1.74E+00	5.18E-07	rs77560159	C/T	8.354
5	174314797	-	-3.58E+00	7.18E-01	5.99E-07	rs62388426	G/A	7.064

### RNA processing

Samples were prepared for sequencing with the IlluminaTruSeq sample preparation kit (Illumina, San Diego, CA) according to the manufacturer’s instructions and were sequenced on a HiSeq2000 machine. An average of 28 million exonic reads per sample was generated. Afterwards, the 49-bp sequenced paired-end reads were mapped to the GRCh37 reference genome [32] with BWA v0.5.9 [33] allowing 2 mismatches in the seed (30 first bases of the read).

#### Gene expression quantification and the MZ discordant model

Genes defined as protein coding in the GENCODE 10 annotation [34] were selected for analysis. RPKM values calculated from the RNA-seq data were root mean transformed and PEER software[35] was used to remove 50 latent factors, after which the data were quantile normalised. Normalised expression data from the LCL tissues were examined in a subset of the samples containing MZ twins discordant for AS with one twin with an AS <10 (control) with a sibling with an ASE score >15 points higher (case). 25 discordant MZ pairs matching the criteria could be identified. A linear mixed effect model was fitted for the scaled exon values of LCL tissue using the R package lmer[36]. The model incorporated ASI, age, BMI, smoking and alcohol consumption of the twins as fixed effects predictors and family structure as a random effect.

### Validation Data

Validation for the GWAS analysis was taken from a current meta-analysis of anxiety disorders. This study, conducted in over 18,000 subjects of European descent, uses two phenotypic strategies to test association with common variants genome-wide that index genetic susceptibility shared across the primary anxiety disorders. The first compared cases with unaffected controls; the second used factor analysis to estimate a quantitative factor score that indexed a common latent risk phenotype[37].

Expression analysis was replicated in an independent dataset of U219 Affy gene expression arrays for 586 twins from the Netherlands Study of Depression and Anxiety (NESDA) and Netherlands Twin Register (NTR) cohorts [38,39] The phenotype used for the NESDA Samples is the Beck Anxiety Inventory (BAI)[40], which is a 27-item questionnaire ranging from 0 to 63 also with high internal consistency (Cronbach’s α = 0.92)[41]. A score of 0–9 refers to normal severity, whereas a score of 10–18 refers to mild severity, a score of 18–29 refers to moderate severity, and a score higher than 29 refers to severe anxiety symptoms. 22 MZ twins highly discordant for BAI were identified for the replication analysis.

### Ethics Statement

The study was approved by the St Thomas’ Hospital Research Ethics Committee (REC reference: EC04/015) in accordance with the St Thomas’ Hospital Local Ethics Committee. All participants in the study provided written informed consent.

## Results

### Heritability of ASI

Heritability analysis of the ASI score using Mx estimated h^2^ as being 0.4445 (CI: 0.3162–0.5548). This is comparable with previous estimate of the heritability of anxiety related traits (see Discussion).

### GWAS study

**Fig 2:**
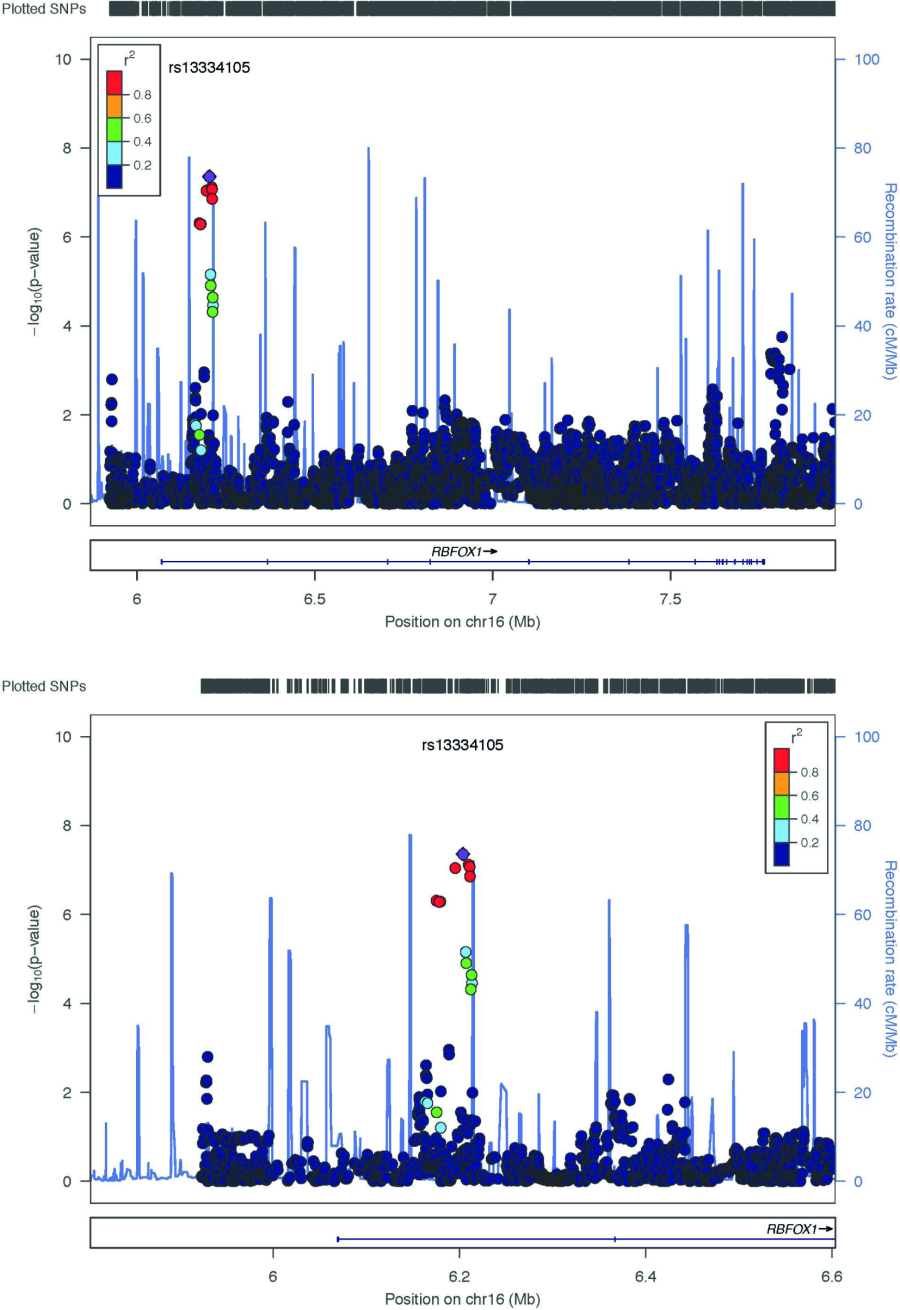
a-b Association plots of the genomewide significant variant rs13334105, showing its location with the RBFOX1 gene region.

**Fig 3:**
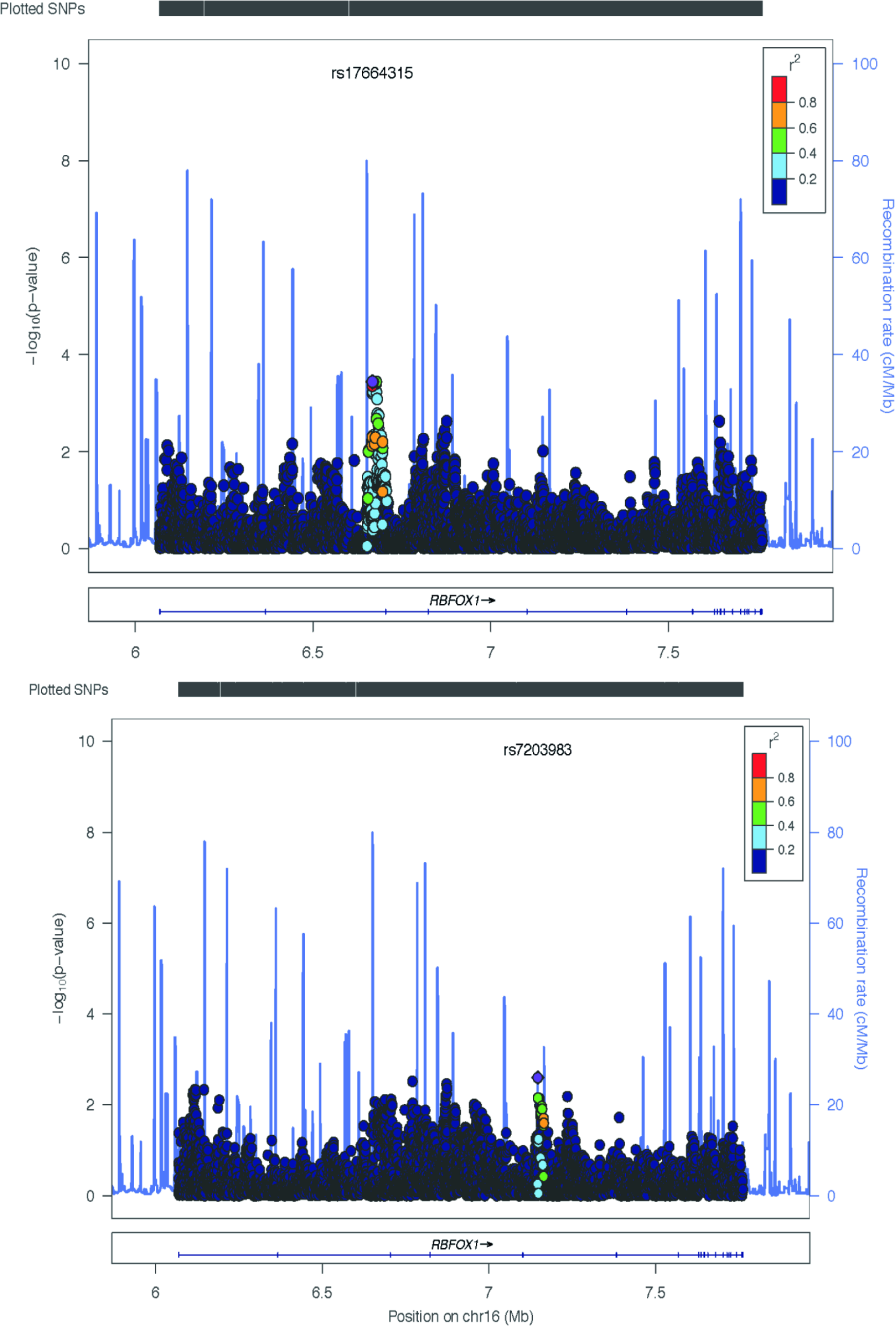
a-b. Otowa *et al*. identified SNPs within the RBFOX1 intronic region with unadjusted p-values of significance, (a) rs17664315 (p = 0.00037) for case-control and (b) rs7203983 (p = 0.00250) for factor scores.

In the AS GWAS analysis, a single variant of genomewide significance (assuming genomewide significance to have an unadjusted p-value < 5x10^-8^[42]) occurred within the coding region of the RBFOX1 gene (rs13334105,p value: 4.39 x10^-8^, MAF = 5.164, T/G). Additionally, several SNPs of suggestive significance also occured within the coding region of the RBFOX1 gene(see Fig 2A and 2B). The significant association in RBFOX1 was compared with the anxiety meta-analysis data (see Data Validation). Although the specific variant rs13334105 was not included in the analysis, both the case control and factor scores approach each identified a SNP within the RBFOX1 intronic region with unadjusted p-values <0.05. The variants were rs17664315 (p = 0.00037) and rs7203983 (p = 0.00250) for case control and factor scores respectively (see Fig 3A and 3B).

### Discordant MZ twin model

**Fig 4:**
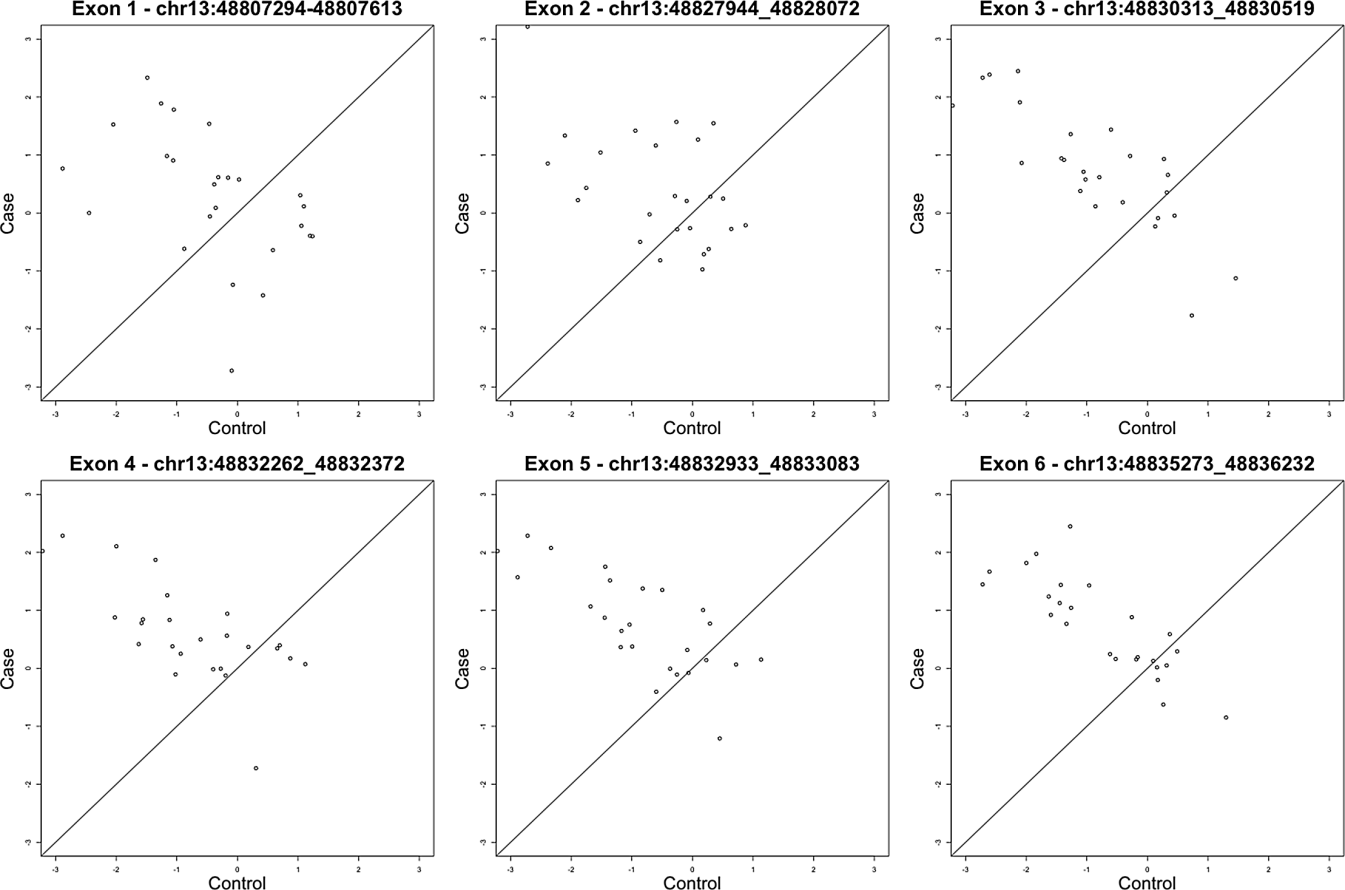
Relative expression of the six ITM2B exon between case and control ASI discordant twins. Significantly higher levels of expression are shown in the twins with higher levels of ASI in the case of exon 5 and exon 6.

An alternative approach to the study of complex traits is the use the twin MZ discordant model [43,44] whereby differences in gene expression can be identified by simultaneously controlling for genotypic variants. Here, we implemented a linear mixed effect model in order to identify differential expression amongst twins discordant for ASI. After correcting for the number of exons interrogated (116,528), an adjusted p-value < 0.05 was observed for two out of six exons (Exon 5 and Exon 6) within the gene ITM2B with a further two exons (Exon 3 and Exon 4) approaching significance. The exons of greatest significance within the model are shown in Table 2. A plot of the raw case control expression data is plotted in Fig 4.

**Table 2:** Top associations of ASI GWAS, showing several SNPs of suggestive significance within the RBFOX1 coding region. rs116206041, the only variant of genomewide significant association is shown in bold.

Chr	Position	Gene	β-value	S.E.	p-value	rs number	Polymorphism	MAF
16	6203400	RBFOX1	-9.03	1.65	4.16E-08	rs116206041	A/G	3.270
16	6198655	RBFOX1	-9.19	1.69	5.69E-08	rs145548005	A/G	1.423
16	6198565	RBFOX1	-9.19	1.69	5.76E-08	rs77503996	A/G	2.068
16	6202428	RBFOX1	-9.19	1.70	6.29E-08	rs142030159	A/C	1.240
16	6197904	RBFOX1	-9.20	1.70	6.32E-08	rs117345367	C/G	1.479
16	6202004	RBFOX1	-9.19	1.70	6.50E-08	rs114364691	C/T	2.028
16	6209355	RBFOX1	-8.77	1.63	7.43E-08	rs141324670	G/T	1.469
16	6210832	RBFOX1	-8.73	1.63	8.44E-08	rs117860441	G/C	1.479
16	6195286	RBFOX1	-9.20	1.72	9.00E-08	rs150201809	T/C	1.331

**Fig 5:**
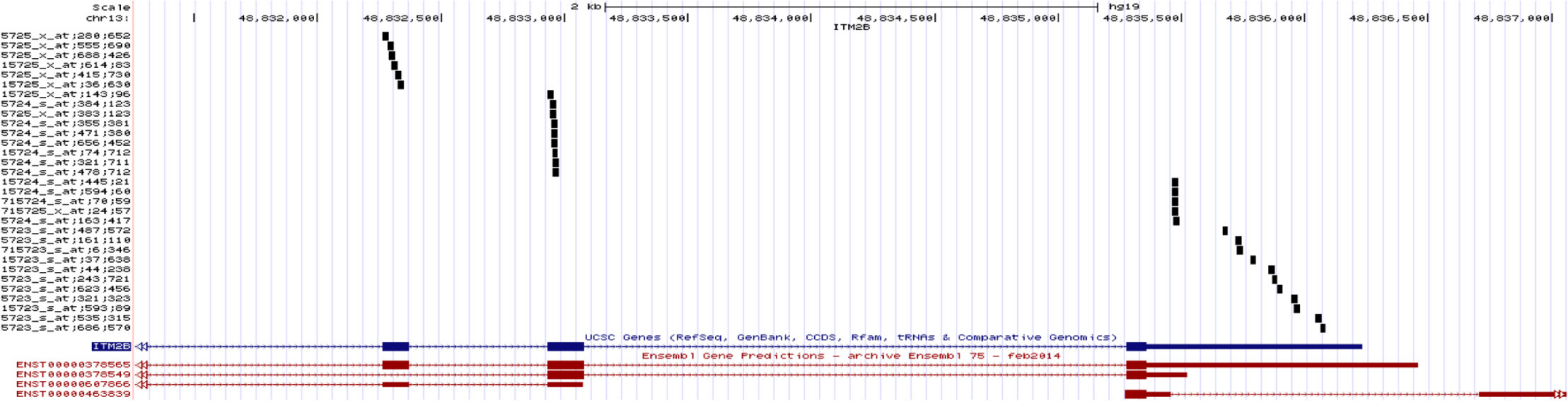
Probe location of exon 4, 5 and 6 of ITM2B in the U219 Affy gene expression array.

**Fig 6:**
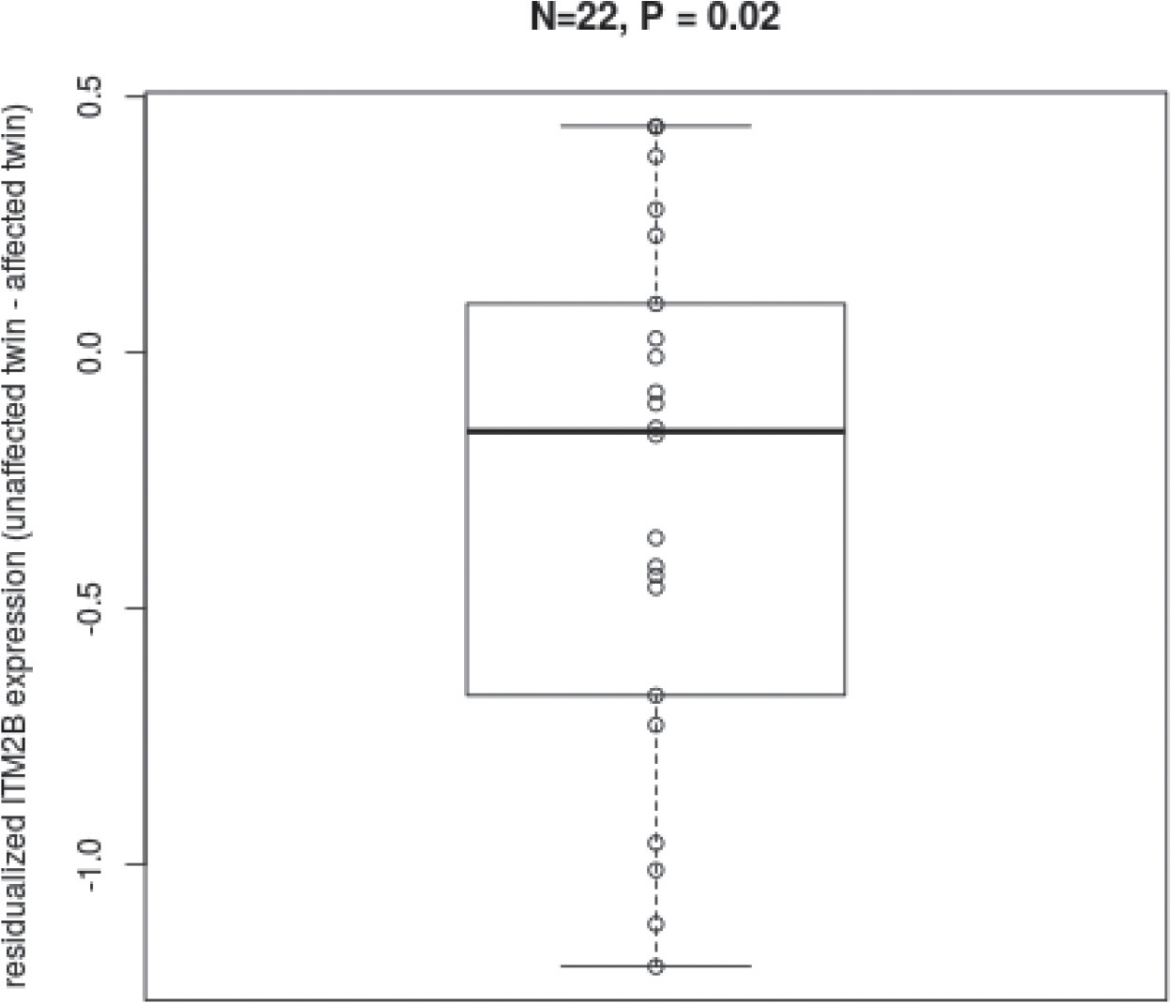
Residualised gene expression showing higher expression levels of the ITM2B gene in the affected twin in comparison to the unaffected sibling in the independent sample.

22 MZ twin pairs discordant for BAI were selected from the U219 Affy gene expression arrays (see Validation Data) In the U219 array, multiple 25 bp probes targeting exon 4 (chr13:48832933–48833083), exon 5 (chr13: 48835273–48836232) and exon 6 (chr13: 48832262–48832372)of ITM2B (see Fig 5) were used to assess gene expression levels. Expression values were residualised by age, sex, BMI, cell counts and technical covariates and the difference between residualized gene expression were computed between pairs. T-tests for this difference on each of the three exons showed p-values of 0.03, 0.3 and 0.002 for exons 4, 5 and 6 respectively. As was the cases with the RNA-seq data, twins with higher anxiety scores in the replication set showed higher expression levels of ITM2B (see Fig 6). The fixed effects incorporated showed no significant association with AS.

## Discussion

Although blood-derived samples may have limited application in the study of neurological function due to the limitations of tissue specificity, previous studies have suggested they can be used as a surrogate tissue for the evaluation of genetic factors in psychiatric disorders[45,46]. Our heritability analysis of AS (h^2^ = 0.445) confirmed previous work suggesting an important genetic contribution to anxiety-related disorders. A study calculating the heritability of AS as a unifactorial construct produced a comparable estimate of h^2^ = 0.45 [47], while in a separate study the heritability of AS was estimated as being 0.48 [48]. The result is also comparable to a previous measure of anxiety using the Anxiety-Related Behaviours Questionnaire (ARBQ), which assesses four anxiety traits [49] and recorded a heritability of 0.52.

Our GWAS analysis also suggested a genetic basis to ASI through the identification of a SNP of genomewide significance and a further eight SNPs of near significance occurring within the coding region of the binding protein, fox-1 homolog (RBFOX1) gene (see Fig 2A and 2B, Table 1). A further GWAS incorporating rare variants (MAF 1–5%) identified another variant of genomewide significance with the RBFOX1 gene.

**Fig 7:**
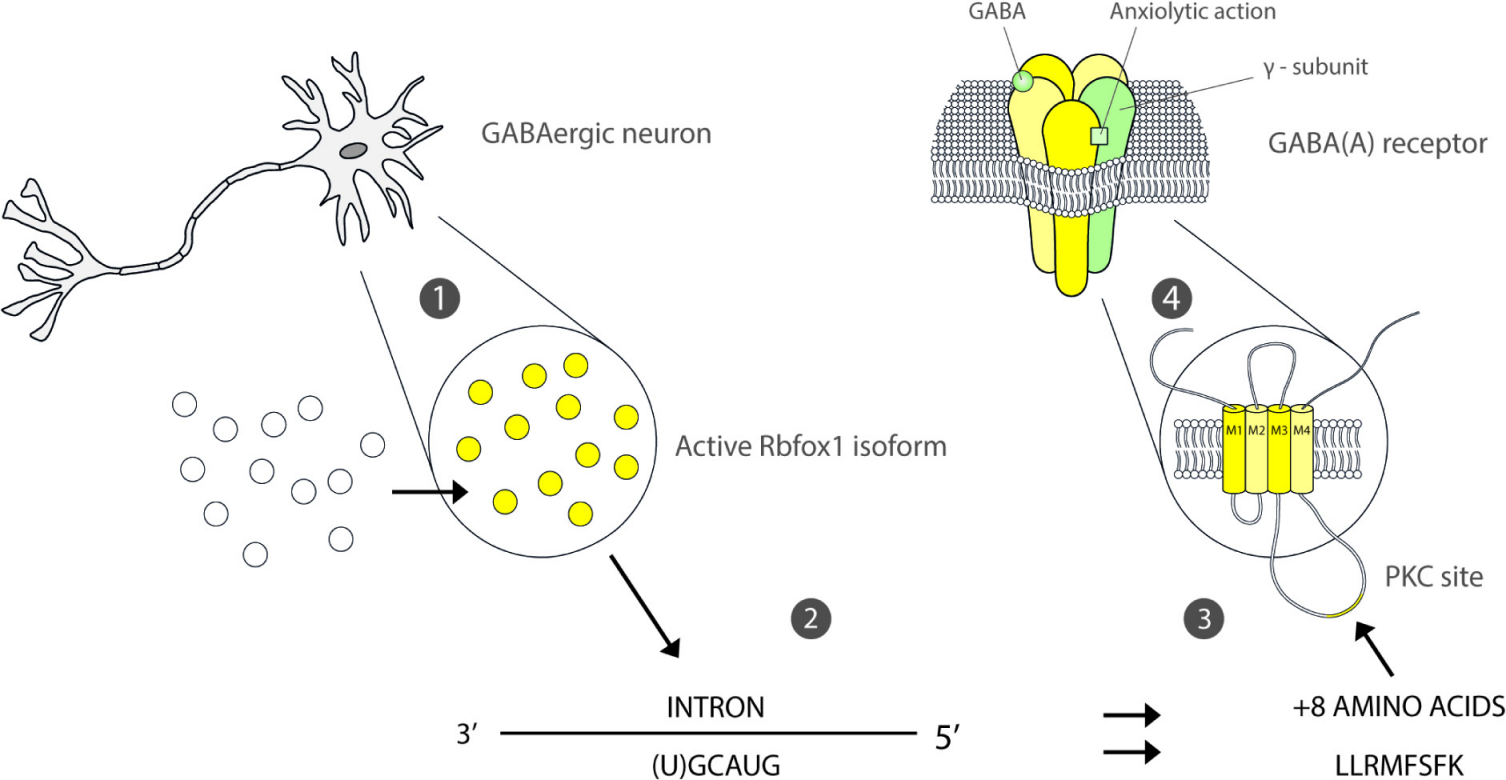
Neuronal depolarisation induces the movement of Fox1 protein from cytoplasm to the nucleus where it is coded by RBFOX1 and increases its capacity to induce splice sites (1). Gabrg2 is regulated by RBFOX1 through the binding of Fox1 protein 1 and Nova near the 5′ and 3′ splice sites of the downstream intron through the targeting of the RNA hexanucleotide UGCAUG (2). Binding to the conserved UGCAUG elements cause an inclusion of 8 amino acids (LLRMFSFK) on the intracellular loop between the M3 and M4 transmembrane domain site. This creates a putative for protein kinase C (PKC) which is a point for serine phosphorylation (3). Phosphorylation of serine alters the γ2 subunit structure of the GABA (A) receptor which controls the regulation of GABAergic neuronal transmission (4).

RBFOX1 (also known as ataxin 2-binding protein 1 (A2BP1) or hexaribonucleotide-binding protein 1 (HRNBP1) codes for the Fox1 protein and regulates alterative splicing which controls the gene expression coordinating neuronal brain activity [50,51]. Abnormalities within the limbic system are the main factor in eliciting anxiety-like symptoms [52,53]. The limbic system is characterised by a significant density of monoaminergic and GABAergic neurons. Patients with GAD have abnormal GABAergic activity resulting from the down-regulation of Gamma-amino butyric acid (GABA-A) receptor, an ionotropic receptor and ligand-gated ion channel[54]. Positive modulators of GABA receptors generally possess anxiolytic (anti-anxiety) activity while negative modulators produce anxiogenic-like effects. The action of anxiolytic drugs work by suppressing this overactive transmission by increasing the GABA-A signal [52]. A probable mechanism by which RBFOX1 influences GABAergic neuronal function is shown in Fig 7 [55]. Immunostaining techniques have shown that there is an increased expression of the active Fox1 isoform in the nucleus following neuronal depolarisation[56]. Fox1 may regulate the action of Gabrg2, a gene which encodes the gamma 2 subunit of GABA(A) receptor, by alternative splicing[57]. Gabrg2 regulation is controlled by the binding of Fox1 near the splice sites of the downstream intron using (U)GCAUG targeting. This sequence inserts 8 amino acids on the intracellular loop between the M3 and M4 transmembrane domain site of the GABA receptor [58–60], creating a putative serine phosphorylation site for protein kinase C. [61,62] Phosphorylation of serine has shown in to alter the tertiary structure of the γ^2^ subunit of the GABA(A) receptor, which has control of the GABA- A receptor function [61–64].

In clinical settings, variations within the RBFOX1 gene have been shown to be associated with autism spectrum disorder (ASD), schizophrenia, epilepsy and attention deficit hyperactivity disorder (ADHD) [65]. This pathology has been clinically evaluated in a case study involving 16-year-old male who has a heterozygous deletion spanning 100.3 kb in the 5' region of the *RBFOX1*. The patient showed speech behavioural abnormalities including anxiety like behaviour towards certain stimuli. This supports a previous study on patients that had copy number variation in the region of *RBFOX1*, which was shown to be associated with a variety of neurodevelopmental and neuropsychiatric disorders [65].

### Differential gene expression between MZ twins discordant for ASI

The MZ twin discordance analysis showed statistically higher significant expression of exons 5 and 6 in the ITM2B (BRI2) gene for the anxiety cohort (see Table 3). ITM2B gene encodes a transmembrane protein which is processed at the C-terminus by furin or furin-like proteases to produce a small secreted peptide which inhibits the deposition of beta-amyloid [66]. A point mutation at the stop coding of ITM2B causes a build up of beta-amyloid plaques which can led to neuronal cell death. ITM2B is therefore strongly associated with the neurodegenerative diseases, such as British and Danish Familial Dementia[67]. The conditions are characterised by significant neuronal loss, a loss of cognitive and memory functions, disruptions to muscle movement and the induction of special rigidity [66,67].

**Table 3:** Differential gene expression of discordant MZs pairs. The two ITM2B exons differentially expressed to a statistically significant degree are shown in bold.

EnsemblID	Gene	Start	Stop	p value	p.value.adj
**ENSG00000136156.7**	**ITM2B**	**48832933**	**48833083**	**1.26E-07**	**0.014696637**
**ENSG00000136156.7**	**ITM2B**	**48835273**	**48836232**	**2.94E-07**	**0.03422515**
ENSG00000136156.7	ITM2B	48832262	48832372	8.11E-07	0.094511195
ENSG00000136156.7	ITM2B	48830313	48830519	9.12E-07	0.106261863
ENSG00000111445.9	RFC5	118463552	118463633	1.97E-06	0.230050387
ENSG00000109618.7	SEPSECS	25158478	25158596	2.18E-06	0.254047203
ENSG00000188735.7	TMEM120B	122213515	122220907	2.25E-06	0.261836002
ENSG00000003756.11	RBM5	50127803	50127884	3.87E-06	0.450813412
ENSG00000067560.6	RHOA	49449837	49450035	4.10E-06	0.477400668
ENSG00000186153.10	WWOX	78466385	78466649	5.70E-06	0.664491474
ENSG00000158716.4	DUSP23	159751943	159752333	5.84E-06	0.680620253
ENSG00000180672.4	AC007362.1	206642948	206644433	6.66E-06	0.775626346
ENSG00000177169.3	ULK1	132398625	132399776	7.03E-06	0.819282771
ENSG00000231903.1	AC079354.5	203043993	203044114	7.20E-06	0.839311775
ENSG00000154277.8	UCHL1	41270004	41270472	7.67E-06	0.893234405
ENSG00000156273.10	BACH1	30714720	30718469	7.70E-06	0.897787193
ENSG00000123485.5	HJURP	234762490	234762556	8.52E-06	0.992346981

The accumulation of beta-amyloids within the amygdala has been shown to drive fear and anxiety-like behavior in transgenic mice models[68]. These observed behaviours are distinct from anxiety symptoms associated with ageing [69]. This model suggests a mechanism for the neuronal connectivity loss that is characteristic of amygdala in GAD [70] and is consistent with studies that demonstrate anxiety-like symptoms being present in neurodegenerative disorders [71]. That said, increased anxiety may also be due to memory loss and physical impairment that are symptomatic of neurodegeneration.

## Conclusion

Our analyses suggest an involvement of the RBFOX1 gene in the development of anxiety-related conditions such as GAD. The GWAS results were supported by an independent study which also identified variants in the RBFOX1 gene which were linked to AS. We further identified two exons of ITM2B as having significantly higher expression levels in individuals with higher levels of AS than their MZ twin, an effect we saw replicated in an independent sample. This also suggests that both genetic and non-genetic variation is crucial to the development of the disorder. Our results may indicate novel etiological mechanisms and paths in the disease development or maintenance of the disease. Further investigation would be necessary to determine whether these variants are specific to AS or whether they are common to more general anxiety disorders.

## Supporting Information

S1 Table Full questionnaire to determine Anxiety Specificity Index (ASI). (DOCX)

## References

[1] Wittchen HU (2002) Generalized anxiety disorder: prevalence, burden, and cost to society. Depress Anxiety 16: 162–171. 12497648 10.1002/da.10065

[2] Tyrer P, Baldwin D (2006) Generalised anxiety disorder. Lancet 368: 2156–2166. 17174708 10.1016/S0140-6736(06)69865-6

[3] Simon NM (2009) Generalized anxiety disorder and psychiatric comorbidities such as depression, bipolar disorder, and substance abuse. J Clin Psychiatry 70 Suppl 2: 10–14. 19371501 10.4088/jcp.s.7002.02

[4] Paris J (2013) Making the DSM-5: concepts and controversies New York: Springer.

[5] Viana AG, Rabian B (2008) Perceived attachment: relations to anxiety sensitivity, worry, and GAD symptoms. Behav Res Ther 46: 737–747. 10.1016/j.brat.2008.03.002 18471800

[6] Lilienfeld SO, Turner SM, Jacob RG (1996) Further comments on the nature and measurement of anxiety sensitivity: A reply to Taylor (1995b). Journal of Anxiety Disorders 10: 411–424.

[7] Naragon-Gainey K (2010) Meta-analysis of the relations of anxiety sensitivity to the depressive and anxiety disorders. Psychol Bull 136: 128–150. 10.1037/a0018055 20063929

[8] Carvalho HW, Andreoli SB, Lara DR, Patrick CJ, Quintana MI, Bressan RA, et al (2014) The joint structure of major depression, anxiety disorders, and trait negative affect. Rev Bras Psiquiatr 36: 285–292. 10.1590/1516-4446-2013-1329 25310205

[9] Waszczuk MA, Zavos HM, Eley TC (2013) Genetic and environmental influences on relationship between anxiety sensitivity and anxiety subscales in children. J Anxiety Disord 27: 475–484. 10.1016/j.janxdis.2013.05.008 23872507 PMC3878378

[10] Allan NP, Capron DW, Raines AM, Schmidt NB (2014) Unique relations among anxiety sensitivity factors and anxiety, depression, and suicidal ideation. J Anxiety Disord 28: 266–275. 10.1016/j.janxdis.2013.12.004 24534564

[11] Olatunji BO, Wolitzky-Taylor KB (2009) Anxiety sensitivity and the anxiety disorders: a meta-analytic review and synthesis. Psychol Bull 135: 974–999. 10.1037/a0017428 19883144

[12] Deacon BJ, Abramowitz JS, Woods CM, Tolin DF (2003) The Anxiety Sensitivity Index—Revised: psychometric properties and factor structure in two nonclinical samples. Behaviour Research and Therapy 41: 1427–1449. 14583412 10.1016/s0005-7967(03)00065-2

[13] Reiss S, Peterson RA, Gursky DM, McNally RJ (1986) Anxiety sensitivity, anxiety frequency and the prediction of fearfulness. Behav Res Ther 24: 1–8. 3947307 10.1016/0005-7967(86)90143-9

[14] Plehn K, Peterson RA (2002) Anxiety sensitivity as a predictor of the development of panic symptoms, panic attacks, and panic disorder: a prospective study. J Anxiety Disord 16: 455–474. 12213039 10.1016/s0887-6185(02)00129-9

[15] Shostak BB, Peterson RA (1990) Effects of anxiety sensitivity on emotional response to a stress task. Behav Res Ther 28: 513–521. 2076089 10.1016/0005-7967(90)90138-9

[16] Rector NA, Szacun-Shimizu K, Leybman M (2007) Anxiety sensitivity within the anxiety disorders: disorder-specific sensitivities and depression comorbidity. Behav Res Ther 45: 1967–1975. 17084380 10.1016/j.brat.2006.09.017

[17] Rodriguez BF, Bruce SE, Pagano ME, Spencer MA, Keller MB (2004) Factor structure and stability of the Anxiety Sensitivity Index in a longitudinal study of anxiety disorder patients. Behav Res Ther 42: 79–91. 14744525 10.1016/s0005-7967(03)00074-3 PMC3272759

[18] Reiss S, Peterson RA, Gursky DM (1988) Anxiety sensitivity, injury sensitivity, and individual differences in fearfulness. Behav Res Ther 26: 341–345. 3214399 10.1016/0005-7967(88)90088-5

[19] Hallmayer J, Cleveland S, Torres A, Phillips J, Cohen B, Torigoe T, et al (2011) Genetic heritability and shared environmental factors among twin pairs with autism. Arch Gen Psychiatry 68: 1095–1102. 10.1001/archgenpsychiatry.2011.76 21727249 PMC4440679

[20] Beckmann H, Franzek E (2000) The genetic heterogeneity of "schizophrenia". World J Biol Psychiatry 1: 35–41. 12607231 10.3109/15622970009150564

[21] Castillo-Fernandez JE, Spector TD, Bell JT (2014) Epigenetics of discordant monozygotic twins: implications for disease. Genome Med 6: 60 10.1186/s13073-014-0060-z 25484923 PMC4254430

[22] Moayyeri A, Hammond CJ, Valdes AM, Spector TD (2013) Cohort Profile: TwinsUK and healthy ageing twin study. Int J Epidemiol 42: 76–85. 10.1093/ije/dyr207 22253318 PMC3600616

[23] Spector TD, Williams FM (2006) The UK Adult Twin Registry (TwinsUK). Twin Res Hum Genet 9: 899–906. 17254428 10.1375/183242706779462462

[24] Grundberg E, Small KS, Hedman AK, Nica AC, Buil A, Keildson S, et al (2012) Mapping cis- and trans-regulatory effects across multiple tissues in twins. Nat Genet 44: 1084–1089. 10.1038/ng.2394 22941192 PMC3784328

[25] Andrew T, Hart DJ, Snieder H, de Lange M, Spector TD, MacGregor AJ (2001) Are twins and singletons comparable? A study of disease-related and lifestyle characteristics in adult women. Twin Res 4: 464–477. 11780939 10.1375/1369052012803

[26] Buil A, Brown AA, Lappalainen T, Vinuela A, Davies MN, Zheng HF, et al (2015) Gene-gene and gene-environment interactions detected by transcriptome sequence analysis in twins. Nat Genet 47: 88–91. 10.1038/ng.3162 25436857 PMC4643454

[27] Neale MC, Hunter MD, Pritikin JN, Zahery M, Brick TR, Kirkpatrick RM, et al (2015) OpenMx 2.0: Extended Structural Equation and Statistical Modeling. Psychometrika. 10.1007/s11336-014-9435-8 PMC4516707 25622929

[28] Akaike H (1987) Factor-Analysis and Aic. Psychometrika 52: 317–332.

[29] The 1000 Genomes Project Consortium, Abecasis GR, Auton A, Brooks LD, DePristo MA, Durbin RM, et al (2012) An integrated map of genetic variation from 1,092 human genomes. Nature 491: 56–65. 10.1038/nature11632 23128226 PMC3498066

[30] Howie BN, Donnelly P, Marchini J (2009) A flexible and accurate genotype imputation method for the next generation of genome-wide association studies. PLoS genetics 5: e1000529 10.1371/journal.pgen.1000529 19543373 PMC2689936

[31] Zhou X, Stephens M (2012) Genome-wide efficient mixed-model analysis for association studies. Nat Genet 44: 821–824. 10.1038/ng.2310 22706312 PMC3386377

[32] Lander ES, Linton LM, Birren B, Nusbaum C, Zody MC, Baldwin J, et al (2001) Initial sequencing and analysis of the human genome. Nature 409: 860–921. 11237011 10.1038/35057062

[33] Li H, Durbin R (2009) Fast and accurate short read alignment with Burrows-Wheeler transform. Bioinformatics 25: 1754–1760. 10.1093/bioinformatics/btp324 19451168 PMC2705234

[34] Harrow J, Frankish A, Gonzalez JM, Tapanari E, Diekhans M, Kokocinski F, et al (2012) GENCODE: the reference human genome annotation for The ENCODE Project. Genome Res 22: 1760–1774. 10.1101/gr.135350.111 22955987 PMC3431492

[35] Parts L, Stegle O, Winn J, Durbin R (2011) Joint genetic analysis of gene expression data with inferred cellular phenotypes. PLoS Genet 7: e1001276 10.1371/journal.pgen.1001276 21283789 PMC3024309

[36] Lamprianou I (2013) Application of single-level and multi-level Rasch models using the lme4 package. J Appl Meas 14: 79–90. 23442329

[37] Hettema JM (2015) Meta-analysis of genome-wide association studies of anxiety disorders. Mol Psychiatry Under Review. 10.1038/mp.2015.197 PMC4940340 26754954

[38] Jansen R, Batista S, Brooks AI, Tischfield JA, Willemsen G, van Grootheest G, et al (2014) Sex differences in the human peripheral blood transcriptome. BMC Genomics 15: 33 10.1186/1471-2164-15-33 24438232 PMC3904696

[39] Wright FA, Sullivan PF, Brooks AI, Zou F, Sun W, Xia K, et al (2014) Heritability and genomics of gene expression in peripheral blood. Nat Genet 46: 430–437. 10.1038/ng.2951 24728292 PMC4012342

[40] Beck AT, Epstein N, Brown G, Steer RA (1988) An inventory for measuring clinical anxiety: psychometric properties. J Consult Clin Psychol 56: 893–897. 3204199 10.1037//0022-006x.56.6.893

[41] De Ayala RJ, Vonderharr-Carlson DJ, Kim D (2005) Assessing the reliability of the beck anxiety inventory scores. Educational and Psychological Measurement 65: 836–850.

[42] Risch N, Merikangas K (1996) The future of genetic studies of complex human diseases. Science 273: 1516–1517. 8801636 10.1126/science.273.5281.1516

[43] Davies MN, Krause L, Bell JT, Gao F, Ward KJ, Wu H, et al (2014) Hypermethylation in the ZBTB20 gene is associated with major depressive disorder. Genome Biol 15: R56 10.1186/gb-2014-15-4-r56 24694013 PMC4072999

[44] Bell JT, Spector TD (2011) A twin approach to unraveling epigenetics. Trends Genet 27: 116–125. 10.1016/j.tig.2010.12.005 21257220 PMC3063335

[45] Mostafavi S, Battle A, Zhu X, Potash JB, Weissman MM, Shi J, et al (2013) Type I interferon signaling genes in recurrent major depression: increased expression detected by whole-blood RNA sequencing. Mol Psychiatry. 10.1038/mp.2013.161 PMC5404932 24296977

[46] Davies MN, Volta M, Pidsley R, Lunnon K, Dixit A, Lovestone S, et al (2012) Functional annotation of the human brain methylome identifies tissue-specific epigenetic variation across brain and blood. Genome Biology 13. 10.1186/gb-2012-13-6-r43 PMC3446315 22703893

[47] Stein MB, Jang KL, Livesley WJ (1999) Heritability of anxiety sensitivity: a twin study. Am J Psychiatry 156: 246–251. 9989561 10.1176/ajp.156.2.246

[48] Burri A, Spector T, Rahman Q (2012) The etiological relationship between anxiety sensitivity, sexual distress, and female sexual dysfunction is partly genetically moderated. J Sex Med 9: 1887–1896. 10.1111/j.1743-6109.2012.02710.x 22462795

[49] Trzaskowski M, Eley TC, Davis OS, Doherty SJ, Hanscombe KB, Meaburn EL, et al (2013) First genome-wide association study on anxiety-related behaviours in childhood. PLoS One 8: e58676 10.1371/journal.pone.0058676 23565138 PMC3614558

[50] Fogel BL, Wexler E, Wahnich A, Friedrich T, Vijayendran C, Gao F, et al (2012) RBFOX1 regulates both splicing and transcriptional networks in human neuronal development. Hum Mol Genet 21: 4171–4186. 10.1093/hmg/dds240 22730494 PMC3441119

[51] Gray JA (1983) A theory of anxiety: the role of the limbic system. Encephale 9: 161B–166B. 6144510

[52] Schienle A, Ebner F, Schafer A (2011) Localized gray matter volume abnormalities in generalized anxiety disorder. Eur Arch Psychiatry Clin Neurosci 261: 303–307. 10.1007/s00406-010-0147-5 20820793

[53] Scheelkruger J, Arnt J, Magelund G, Olianas M, Przewlocka B, Christensen AV (1980) Behavioral Functions of Gaba in Basal Ganglia and Limbic System. Brain Research Bulletin 5: 261–267.

[54] Farb DH, Ratner MH (2014) Targeting the modulation of neural circuitry for the treatment of anxiety disorders. Pharmacol Rev 66: 1002–1032. 10.1124/pr.114.009126 25237115

[55] Gehman LT, Stoilov P, Maguire J, Damianov A, Lin CH, Shiue L, et al (2011) The splicing regulator Rbfox1 (A2BP1) controls neuronal excitation in the mammalian brain. Nat Genet 43: 706–711. 10.1038/ng.841 21623373 PMC3125461

[56] Lee JA, Tang ZZ, Black DL (2009) An inducible change in Fox-1/A2BP1 splicing modulates the alternative splicing of downstream neuronal target exons. Genes Dev 23: 2284–2293. 10.1101/gad.1837009 19762510 PMC2758739

[57] Zhang C, Frias MA, Mele A, Ruggiu M, Eom T, Marney CB, et al (2010) Integrative modeling defines the Nova splicing-regulatory network and its combinatorial controls. Science 329: 439–443. 10.1126/science.1191150 20558669 PMC3412410

[58] Dredge BK, Darnell RB (2003) Nova regulates GABA(A) receptor gamma2 alternative splicing via a distal downstream UCAU-rich intronic splicing enhancer. Mol Cell Biol 23: 4687–4700. 12808107 10.1128/MCB.23.13.4687-4700.2003 PMC164843

[59] Jin Y, Suzuki H, Maegawa S, Endo H, Sugano S, Hashimoto K, et al (2003) A vertebrate RNA-binding protein Fox-1 regulates tissue-specific splicing via the pentanucleotide GCAUG. EMBO J 22: 905–912. 12574126 10.1093/emboj/cdg089 PMC145449

[60] Nakahata S, Kawamoto S (2005) Tissue-dependent isoforms of mammalian Fox-1 homologs are associated with tissue-specific splicing activities. Nucleic Acids Res 33: 2078–2089. 15824060 10.1093/nar/gki338 PMC1075922

[61] Kofuji P, Wang JB, Moss SJ, Huganir RL, Burt DR (1991) Generation of two forms of the gamma-aminobutyric acidA receptor gamma 2-subunit in mice by alternative splicing. J Neurochem 56: 713–715. 1846404 10.1111/j.1471-4159.1991.tb08209.x

[62] Krishek BJ, Xie X, Blackstone C, Huganir RL, Moss SJ, Smart TG (1994) Regulation of GABAA receptor function by protein kinase C phosphorylation. Neuron 12: 1081–1095. 8185945 10.1016/0896-6273(94)90316-6

[63] Poisbeau P, Cheney MC, Browning MD, Mody I (1999) Modulation of synaptic GABAA receptor function by PKA and PKC in adult hippocampal neurons. J Neurosci 19: 674–683. 9880588 10.1523/JNEUROSCI.19-02-00674.1999 PMC6782188

[64] Crestani F, Lorez M, Baer K, Essrich C, Benke D, Laurent JP, et al (1999) Decreased GABAA-receptor clustering results in enhanced anxiety and a bias for threat cues. Nat Neurosci 2: 833–839. 10461223 10.1038/12207

[65] Zhao WW (2013) Intragenic deletion of RBFOX1 associated with neurodevelopmental/neuropsychiatric disorders and possibly other clinical presentations. Molecular Cytogenetics 6. 10.1186/1755-8166-6-26 PMC3766065 23822903

[66] Kilger E, Buehler A, Woelfing H, Kumar S, Kaeser SA, Nagarathinam A, et al (2011) BRI2 protein regulates beta-amyloid degradation by increasing levels of secreted insulin-degrading enzyme (IDE). J Biol Chem 286: 37446–37457. 10.1074/jbc.M111.288373 21873424 PMC3199491

[67] Kim J, Miller VM, Levites Y, West KJ, Zwizinski CW, Moore BD, et al (2008) BRI2 (ITM2b) inhibits Abeta deposition in vivo. J Neurosci 28: 6030–6036. 10.1523/JNEUROSCI.0891-08.2008 18524908 PMC2586000

[68] Espana J, Gimenez-Llort L, Valero J, Minano A, Rabano A, Rodriguez-Alvarez J, et al (2010) Intraneuronal beta-amyloid accumulation in the amygdala enhances fear and anxiety in Alzheimer's disease transgenic mice. Biol Psychiatry 67: 513–521. 10.1016/j.biopsych.2009.06.015 19664757

[69] Bedrosian TA, Herring KL, Weil ZM, Nelson RJ (2011) Altered temporal patterns of anxiety in aged and amyloid precursor protein (APP) transgenic mice. Proc Natl Acad Sci U S A 108: 11686–11691. 10.1073/pnas.1103098108 21709248 PMC3136261

[70] Etkin A, Prater KE, Schatzberg AF, Menon V, Greicius MD (2009) Disrupted Amygdalar Subregion Functional Connectivity and Evidence of a Compensatory Network in Generalized Anxiety Disorder. Archives of General Psychiatry 66: 1361–1372. 10.1001/archgenpsychiatry.2009.104 19996041 PMC12553334

[71] Ferretti L, McCurry SM, Logsdon R, Gibbons L, Teri L (2001) Anxiety and Alzheimer's disease. Journal of Geriatric Psychiatry and Neurology 14: 52–58. 11281317 10.1177/089198870101400111

