# Supplementary material for: Generalised Anxiety Disorder – A Twin Study of Genetic Architecture, Genome-Wide Association and Differential Gene Expression
Source: PLoS One. 2015 Aug 14;10(8):e0134865. doi: 10.1371/journal.pone.0134865 (PMC4537268; doi:10.1371/journal.pone.0134865)
Supplement: S1 Table — (DOCX) [file pone.0134865.s001.docx]

ASI Table

| **Please rate the extent to which each statement applies to you:** |  |
| --- | --- |
| It is important to me not to appear nervous | 0 1 2 3 4 |
| When I cannot keep my mind on a task, I worry that I might be going crazy | 0 1 2 3 4 |
| It scares me when I feel “shaky” (trembling) | 0 1 2 3 4 |
| It scares me when I feel faint | 0 1 2 3 4 |
| It is important to me to stay in control of my emotions | 0 1 2 3 4 |
| It scares me when my heart beats rapidly | 0 1 2 3 4 |
| It embarrasses me when my stomach growls | 0 1 2 3 4 |
| It scares me when I am nauseous | 0 1 2 3 4 |
| When I notice that my heart is beating rapidly, I worry that I might have a heart attack | 0 1 2 3 4 |
| It scares me when I become short of breath | 0 1 2 3 4 |
| When my stomach is upset, I worry that I might be seriously ill | 0 1 2 3 4 |
| It scares me when I am unable to keep my mind on a task | 0 1 2 3 4 |
| Other people notice when I feel shaky | 0 1 2 3 4 |
| Unusual body sensations scare me | 0 1 2 3 4 |
| When I am nervous, I worry that I might be mentally ill | 0 1 2 3 4 |
| It scares me when I am nervous | 0 1 2 3 4 |
